# Use of failure mode and effects analysis for risk analysis of investigational drug products management in clinical trials

**DOI:** 10.1093/ajhp/zxaf308

**Published:** 2025-11-10

**Authors:** Bin Zhang, Mingming Song, Ziwei Wang, Ying Zhou, Xiaocong Pang

**Affiliations:** Department of Pharmacy, Peking University First Hospital, Beijing, China; Department of Pharmacy, Peking University First Hospital, Beijing, China; Drug Clinical Trial Institution, Peking University First Hospital, Beijing, China; Department of Pharmacy, Peking University First Hospital, Beijing, China; and Drug Clinical Trial Institution, Peking University First Hospital, Beijing, China; Department of Pharmacy, Peking University First Hospital, Beijing, China

**Keywords:** FMEA, investigational drug product, IDS pharmacy, participant safety, risk analysis

## Abstract

**Background:**

The purpose of this study is to prospectively analyze the entire process of managing investigational drug products (IP) in the investigational drug service (IDS) pharmacy, explore the safety risks and corrective actions in the management of IP, and ensure the safety of participants and the quality of clinical trials.

**Methods:**

Failure mode and effects analysis (FMEA) was conducted at a tertiary general hospital. By analyzing the risk priority number (RPN) of each failure mode, failure modes with significant impacts on participant safety, data integrity, and regulatory compliance in clinical trials were identified, and implementation priorities were determined. Corresponding corrective actions were then formulated.

**Results:**

A total of 44 failure modes were identified, with 31 classified as high-risk. The causes of these failure modes were primarily related to factors such as IDS pharmacists failing to strictly review documents, noncompliance with standard operating procedures, and investigators’ lack of familiarity with clinical trial protocols. Finally, 5 general and 10 specific corrective actions for particular steps were proposed. Among the top 10 RPN scores, 60.0% were related to the drug dispensing process, and 62.5% of the highest severity scores (5 points) were also associated with this step. The most critical general actions formulated by the team included creating detailed flowcharts for each operational step, developing pocket guides, and enhancing training for pharmacists and investigators.

**Conclusion:**

Through the active participation of IDS pharmacy staff, FMEA successfully identified and prioritized potential failure modes in the IDS pharmacy management process. Corrective actions were proposed to ensure participant safety and continuously improve the quality of clinical trials and IP management.

Key Points

- This study, through the active participation of investigational drug services (IDS) pharmacists, successfully identified potential failure modes in the IDS pharmacy management process using failure mode and effects analysis (FMEA).
- The study identified a total of 44 failure modes in the IDS pharmacy management process and determined 31 high-risk failure modes, leading to proposal of 5 general corrective actions and 10 step-specific corrective actions.
- Among the top 10 risk priority number (RPN) scores, 60.0% were related to the drug dispensing process, and 62.5% of the highest-severity RPN scores (5 points) were also associated with this step.

Investigational drug services (IDS), also referred to as investigational drug product (IP) management, by a clinical research pharmacy plays a vital part in the medication-use process for products used in clinical trials. Responsibilities include, but are not limited to, product receipt, storage, dispensing, and return.^1-4^ Anecdotally, the challenges faced by the IDS pharmacy in IP management for clinical trials are on the rise, along with the associated demands. IDS pharmacists in academic listserver posts and networking focus groups corroborate the increasing difficulty in making accommodations for growing clinical and administrative demands, such as complex preparation (eg, high-risk compounding, blinding), extensive sponsor training modules, stringent storage and documentation requirements, and use of interactive Web response systems.^5^

With the increasing difficulty and complexity of IP management, medication errors are inevitable in the process of clinical trials. According to a report on medication safety in cancer clinical trials, the processes where the errors originated were prescribing (47%), administration (10%), dispensing (6%), and monitoring (5%).^6^ Therefore, the occurrence of such a high rate of medication errors reminds us that the prevention of medication errors in IP management is an essential topic that urgently needs attention.

The IDS pharmacy staff (hereinafter referred to as “pharmacy staff”) recognize the significance of properly managing and accurately documenting the IP administration process. The ultimate goals of IP management are to improve patient safety, improve efficiency, and provide robust clinical data that allow new and innovative medications to reach the patients who need them. Failure to accurately document IP accountability can undermine the validity of clinical study data, which could spur sponsors to halt a site’s participation in current or future studies and result in a loss of local patients’ access to clinical studies.^1^ Moreover, medication errors in any link of IPs management can affect the trial quality to varying degrees. In severe cases, they may even place participant safety at risk. Recently, the International Council for Harmonization of Technical Requirements for Pharmaceuticals for Human Use (ICH) published ICH GCP E6 (R3). This guideline encourages a risk-based and proportionate approach to the conduct of a clinical trial. IP management should be arranged and conducted in accordance with applicable regulatory requirements, and safeguards should be in place to ensure product integrity, product use per protocol, and participant safety.^7^ However, there is a lack of specific practices and relevant research on the risk of IPs management for clinical trials. Hence, risk identification, evaluation, and control in the IP management process in clinical trials represent a critical issue that necessitates prompt resolution and comprehensive consideration.

Failure mode and effects analysis (FMEA) is a proactive, forward-directed method to identify potential failures and hidden dangers in a system or process, analyze their causes and effects, propose preventive measures, and reduce the occurrence rate and severity of failures.^8-10^ As an important quality assurance tool, FMEA is frequently applied in industries such as the aviation, aerospace, nuclear power, and automotive industries.^8^ Although the concept of FMEA originated in other industries, it is now widely used in the healthcare field to analyze complex processes.^9^ Several previous studies have shown that FMEA helps identify, analyze, and reduce risks related to pharmacies.^11-14^ However, such research has not been conducted in the area of IPs management in clinical trials.

Therefore, the objective of this study is to innovatively apply the FMEA tool to identify, evaluate, and analyze risks within the IP management process in clinical trials. Additionally, corresponding corrective actions will be formulated. The ultimate aim is to minimize the occurrence of medication errors in clinical trials, thereby safeguarding the safety of participants and ensuring the high-quality data integrity of clinical trials.

## Methods

### Study setting

To comprehensively support clinical research conducted across outpatient and inpatient departments in our multicampus hospital, we have established 4 IDS pharmacies staffed by 4 full-time IDS pharmacy staff and 4 part-time IDS pharmacy staff (3 of whom also undertake quality control for clinical trial projects and 1 of whom additionally serves as a clinical pharmacist). These pharmacies collectively manage over 200 active drug clinical trials.

Our IDS pharmacies adhere to a unified set of policies and standard operating procedures (SOPs). We utilize a hospital-purchased clinical trial management system (CTMS) to manage IPs, enabling IDS pharmacy staff to document all IP-related activities—including receipt, storage, dispensing, and return—within the system. The CTMS also facilitates monthly inventory reconciliation and workload tracking for the pharmacies. Notably, the CTMS does not support IP ordering; instead, IDS pharmacy staff typically coordinate with clinical research associates (CRAs) to place orders with suppliers. While all IP management records are traceable in the CTMS, hard copies are also printed and archived.

Interactive response technology (IRT) (eg, interactive voice or Web response systems [IVRS/IWRS]) is provided by the sponsor of each project (IRT is generally not provided for nonrandomized trials) to randomly assign participants to study groups and assign kit numbers. Typically, IRT can automatically order IPs.

The investigator prescribes the IP through CTMS. The clinical research coordinator (CRC) then brings the prescription and randomization form (if applicable) to the IDS pharmacy to obtain the IP. The pharmacy staff reviews prescription compliance with the study protocol and dispenses the IP accordingly, recording the transaction in the CTMS. The CRC subsequently administers the IP to the participant or transfers it to the study nurse for preparation.

This research project was conducted at the IDS pharmacy of our hospital from September 2024 to February 2025.

### Team establishment

To ensure the credibility of the evaluation results, we considered the risk management tips proposed by the American Society for Healthcare Risk Management and the Institute for Safe Medication Practices Canada (ISMP Canada). The FMEA team comprises frontline pharmacy staff and management personnel. This is because they have a clear understanding of daily work processes, rich practical experience, and certain perspectives on risk management.^8,15^ The cross-sectional research team of this study consists of 4 frontline pharmacy staff and 1 person in charge of the IDS pharmacy.

### Study design

This study reviewed every complex step of IP management in clinical trials, from receipt to return to the sponsor. This study was conducted in 4 stages.

#### Stage 1

Four frontline pharmacy staff and a leader of the clinical trial pharmacy were chosen for interviews and brainstorming to analyze and determine the key steps in IP management, from receipt to return, and drew a flowchart. Meanwhile, the potential failure risks at each step that could lead to medication errors in clinical trials were listed. Finally, a 5-point scale was developed for scoring the severity (S), occurrence probability (O), and detectability (D) of failure modes.

#### Stage 2: create and complete the FMEA questionnaire

Each team member was trained on the FMEA method and the questionnaire evaluation criteria. All team members independently completed the assessment of the S, O, and D of each failure mode by assigning a score of 1 to 5. The final results presented here are the median values of S, O, and D for each failure mode.

#### Stage 3: calculate the risk priority number (RPN) value based on each score (RPN = S × O × D), and arrange the RPN values in descending order to determine the priority of failure modes

The RPN is in numerical form, with a value range of 1 to 125. The risk at each step and the degree of control measures required increase as the number increases.

#### Stage 4

According to the ISMP guidelines, we selected any potential failure modes with a severity score of 5 and the top 70% of those with the highest criticality scores for further processing^8^ and determined them as high-risk potential failures. Corrective actions and recommendations were established for these failure modes to prevent their occurrence. Data analysis was conducted using Microsoft Oﬃce Excel 2013.

## Results

**Figure 1.**
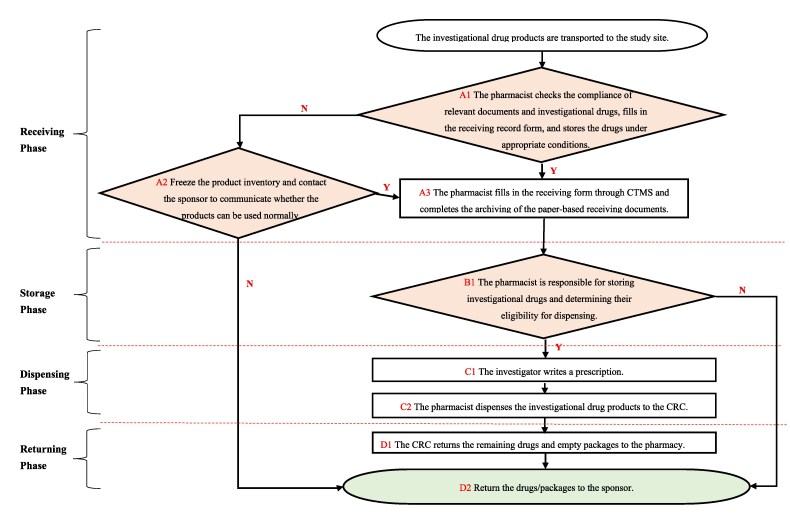
Investigational drug product management process and key steps by the investigational drug services pharmacy. CTMS indicates clinical trial management system; CRC, clinical research coordinator.

IP management at the study site was mainly divided into 4 phases: receipt, storage, dispensing, and return. The entire process consisted of 9 major steps from receipt to return in the IDS pharmacy (Figure 1). This study adopted the brainstorming technique to determine the scoring criteria for the S, O, and D of each potential failure in IP management (Table 1). Subsequently, the prospective risk analysis yielded a total of 44 potential failure modes distributed throughout the whole process, along with 6 main causes. The S, O, and D values were calculated for each failure mode, with the RPN ranging from 5 to 45 (Table 2). Furthermore, a total of 31 potential failure modes posing high risk were identified, of which approximately 29, representing the top 70% of the highest RPN composite scores (with a cut-off value of RPN = 16), were highlighted. Additionally, there were 2 high-risk potential failures with lower RPN scores but a severity score of 5. Finally, 5 general and 10 step-specific corrective actions were proposed, as shown in Table 2.

**Table 1.** Scoring Criteria for Severity, Occurrence Probability, and Detectability of Failure Modes

	Qualitative assessment	Score
**Severity**		
The impact on the management of investigational drug product and the participant is negligible.	Negligible	1
Errors occur in the records of investigational drug product management due to certain reasons, but their authenticity can be verified through other materials, and it does not affect the participant’s safety and the reliability of trial data.	Minor	2
May cause violations of policies, SOPs, or protocols, but does not affect the participant’s safety and the reliability of trial data.	Moderate	3
May result in serious errors such as violations or major protocol deviations, affecting the reliability of trial data but not affecting the participant’s safety.	Severe	4
May lead to the participant having no medicine available, may cause the participant to take the wrong medicine, and may affect the participant’s safety.	Very severe	5
**Occurrence**		
Impossible to occur	Impossible	1
1 time ≤ occurs per year < 4 times	Low	2
1 time ≤ occurs per quarter < 3 times	Medium	3
≥1 to <4 times/month	High	4
Occurs ≥1 time per week	Extremely high	5
**Detectability**		
Extremely easy to be detected.	Extremely easy	1
It is very easy to find the occurrence of such medication errors during routine self-checks at work.	Easy	2
It is very likely to detect the occurrence of such medication errors during routine self-checks at work.	Moderate	3
It is possible to detect the occurrence of such medication errors during routine self-checks at work.	Difficult	4
It is almost impossible to detect the occurrence of such medication errors during routine self-checks at work.	Impossible	5

**Table 2.** Evaluation, Ranking of Risk Priority Numbers for All Failure Modes, and Corrective Actions

No.	Phase	Step	Element	Potential failure mode	S	0	D	RPN	Rank	Corrective actions	
										General	Step specific
1	Receipt phase	The pharmacist checks the compliance of relevant documents and investigational drugs, fills in the receiving record form, and stores the drugs under appropriate conditions. (A1)	Shipment documents	The pharmacist failed to notice the sponsor’s lack of an analysis certificate for investigational product drugs.^a^	3	2	4	24	11	Create detailed operational flowcharts for each step, listing the documents to review, check items, and recording requirements. Display these prominently in the work area for easy reference by pharmacists during operations. Make pocket guides for investigators and CRCs, outlining prescription issuance, dispensing, return processes and step-by-step key points. Strengthen pharmacist training. Regularly summarize and resolve issues in investigational drug product management, raise awareness, and prevent error recurrence. The institution should Regularly organize clinical research training for investigators. Before the study starts, communicate with the CRA to clarify the requirements of sponsors, vendors and logistics providers in investigational drug product management.	When receiving drugs, pharmacists should do double-checks per the flowchart. For special issues, check SOP and report to the supervisor.
2				The pharmacist failed to notice the mismatch between the analysis certificate provided by the sponsor and the batch number of the shipped products.^a^	3	2	4	24	11		
3				The pharmacist failed to notice the absence of a temperature monitoring device during product shipment.^a^	5	1	1	5	44		
4				The pharmacist failed to notice the lack of a calibration certificate for the temperature monitoring device used during product shipment.^a^	4	2	3	24	11		
5			Investigational drug product	The pharmacist failed to notice the temperature excursion during shipment.^a^	5	1	2	10	37		
6				The pharmacist failed to note that the temperature-controlled period during transit was shorter than the time logged on the express bill.^a^	4	2	3	24	11		
7				The pharmacist failed to notice the damaged product packaging or broken vials.	3	2	2	12	30		
8				The pharmacist failed to notice the mismatch between the information (eg, product name, batch number, quantity, kit number) of the shipped investigational drugs and the packing slip.^a^	3	2	3	18	24		
9			IDS pharmacist	Before shipping products to the study site, pharmacists aren’t authorized by the principal investigator.	3	2	2	12	30		
10				The pharmacist didn’t store investigational drugs as expected.^a^	5	2	2	20	22		
11				The shipment arrived in poor condition (eg, physical damage or temperature excursion), but the pharmacist didn’t notify the sponsor and PI immediately.^a^	4	2	3	24	11		
12				The pharmacist didn’t download, print, or file the shipping temperature records.	3	1	3	9	38		
13				The pharmacist didn’t sign documents like the product receiving record, packing list, and express delivery note for confirmation.^a^	2	3	4	24	11		
14				The pharmacist didn’t use IRT to acknowledge the investigational drug shipment as “received in good condition.”	2	2	3	12	30		
15		Freeze the product inventory and contact the sponsor to communicate whether the products can be used normally. (A2)	Sponsor	The drugs had temperature excursions, but the pharmacist failed to notice the sponsor’s lack of adequate documentation for acceptable deviations.^a^	4	2	3	24	11		Upon receiving unqualified drugs, pharmacists should quickly put them in the quarantine area and mark the storage spot.
16			IDS pharmacist	The shipment arrived in sub-standard condition (eg, physical damage or temperature excursion), but the pharmacist didn’t make corresponding transactions in IRT and CTMS to mark the product as damaged or quarantined.	4	2	4	32	6		
17		The pharmacist fills in the receiving form through CTMS and completes the archiving of the paper-based receiving documents. (A3)	IDS pharmacist	The pharmacist input incorrect product information (eg, product name, specification, packaging, quantity, batch number, expiration date, kit number) in CTMS.^a^	2	3	4	24	11		Information entry should be double-checked. At the end of each trial, archived documents should be carefully reviewed.
18				The pharmacist put the receipt documents into another study’s file.	1	2	4	8	42		
19	Storage phase	The pharmacist is responsible for storing investigational drugs and determining their eligibility for dispensing. (B1)	IDS pharmacist	The pharmacist didn’t conduct monthly inventories of the products stored in the pharmacy.	3	1	4	12	30		Create a temperature excursion form，pharmacists must follow its steps precisely for affected products.
20				The counted product quantity didn’t match the quantity in CTMS.^a^	4	2	3	24	11		
21				The pharmacist didn’t check the product storage conditions in the temperature monitoring system every weekday.^a^	3	2	3	18	24		
22				The pharmacist didn’t quarantine products with temperature deviations or expired ones in CTMS.^a^	4	2	3	24	11		
23				The pharmacist failed to physically quarantine products with temperature deviations or expired ones from the usable inventory.^a^	4	2	3	24	11		
24				The pharmacist didn’t promptly notify the sponsor and the PI of the temperature deviation during product storage.	3	1	3	9	38		
25				The temperature monitoring devices and refrigerators in the pharmacy weren’t calibrated as scheduled.	4	1	2	8	42		
26	Dispensing phase	The investigator writes a prescription. (C1)	Investigator	When calculated based on the usage and dosage, the quantity of the prescribed drugs can’t cover the participant’s visit window period.^a^	4	2	4	32	6		Pharmacists should strengthen the review of prescription plan compliance. For each enrolled participant, a dosing plan should be prepared to facilitate quick and accurate prescription verification during drug dispensing.
27				The type, usage, dosage or treatment course of investigational drugs in the prescription didn't follow the protocol.^a^	4	3	3	36	5		
28				The information on the prescription (eg, randomization number, visit name, drug name, kit number, quantity, usage, dosage, batch number) didn’t match the randomization form.^a^	5	3	3	45	1		
29				The investigator didn’t sign and confirm the randomization form or the prescription.^a^	3	3	2	18	24		
30				The blinded investigator prescribed nonblinded investigational drugs.^a^	4	1	4	16	27		
31		The pharmacist dispenses the investigational drug products to the CRC. (C2)	CRC	The CRC in charge of drug collection wasn’t authorized by the PI.	3	3	1	9	38		Pharmacists should send inventory reminder emails to CRAs for projects with low inventory during monthly inventory checks. Pharmacists should mark in advance which projects require randomization forms for each drug dispensing before drug distribution for projects. Embed a barcode technology in the CTMS. Pharmacists need to scan the drug barcodes for double - checking when dispensing drugs. Also, write the participant's number, abbreviated name, visit period, and date on the drug packaging.
32			Investigational drug product	During the follow-up of participants in the open-label trial, it was found that the investigational drug inventory was insufficient for the medication period.^a^	5	3	3	45	1		
33				During the follow-up of participants in the randomized double-blinded trial, the IRT system failed to randomize the drugs to be used.^a^	5	2	2	20	22		
34			IDS pharmacist	For trials with kit numbers assigned via IRT system, pharmacists dispensed IPs solely based on prescriptions without access to the randomization form. ^a^	5	2	4	40	3		
35				The nonblinded pharmacist dispensed drugs to the blinded CRC.^a^	4	1	4	16	27		
36				The pharmacist dispensed the drugs incorrectly.^a^	5	2	4	40	3		
37	Return phase	The CRC returns the remaining drugs and empty packages to the pharmacy. (D1)	CRC	The CRC responsible for drug return wasn’t authorized by the PI.	3	3	1	9	38		Pharmacists should strengthen the verification of the return form, the number of returned drugs, and packaging, and calculate the medication compliance together with the CRC.
38				The quantities of drugs and the number of drug packages to be returned stated on the drug return form recorded by the CRC didn’t match the actual retrieved quantities.^a^	4	4	2	32	6		
39				On the drug return form recorded by the CRC, the recorded quantity of the drug used by the participant is inconsistent with the actual medication intake.^a^	4	4	2	32	6		
40				The CRC didn’t record the participant’s medication information on the drug return form.	3	4	1	12	30		
41				The CRC didn’t return the remaining investigational drugs and used empty packaging according to the visit dates stipulated in the study protocol.^a^	1	4	4	16	27		
42		Return the drugs/packages to the sponsor. (D2)	IDS pharmacist	The drug return form generated by the pharmacist through the CTMS shows discrepancies in information, like the batch number and quantity of the actually returned drugs.^a^	3	3	3	27	10		
43				The nonblinded pharmacist returned the drugs to the blinded CRA.	4	1	3	12	30		
44			CRA	The information (eg, batch number and quantity) on the drug return form prepared by the CRA didn’t match the actually returned drugs.	2	3	2	12	30		

Abbreviations: CRA, clinical research associate; CRC, clinical research coordinator; CTMS, clinical trial management system; D, detectability; IDS, investigational drug service; IRT, interactive response technology; O, occurrence; PI, principal investigator; RPN, risk priority number; S, severity.

^a^High-risk failure mode.

### Failure modes

Analyses conducted in this study indicated that the potential failure modes with the highest RPNs were as follows: “The information on the prescription (eg, randomization number, visit name, drug name, kit number, quantity, usage, dosage, batch number) didn’t match the randomization form” (RPN = 45) and “During the follow-up of participants, it was found that the IP inventory was insufficient for the medication period” (RPN = 45). Apart from those modes, “For trials with kit numbers assigned via IRT system, pharmacy staff dispensed IPs solely based on prescriptions without access to the randomization form” (RPN = 40) and “The pharmacy staff dispensed the drugs incorrectly” (RPN = 40) were also acknowledged as the greatest points of concern. Apart from the aforementioned 4 high-risk failure modes with an S value of 5, there were an additional 4 items: “The pharmacy staff didn’t store IPs as expected” (RPN = 20), “During the follow-up of the participants in the randomized double-blinded trial, the IRT system failed to randomize the drugs that should be used” (RPN = 20), “The pharmacy staff failed to notice the temperature excursion during shipment” (RPN = 10), and “The pharmacy staff failed to notice the absence of a temperature monitoring device during product shipment” (RPN 5). Among the top 10 failure modes, 60% (6/10) originated from the dispensing phase. Of all high-risk failure modes, 41.9% (13/31) were associated with the receiving phase, followed by 32.3% (10/31) in the dispensing phase.

### Causes

The primary causes of top failure modes can be summarized into the following 6 aspects: (1) Pharmacy staff did not strictly review documents, did not strictly follow SOPs, or were negligent in their work; (2) Investigators were not familiar with the protocol, or had a weak awareness of ethics and good clinical practice (GCP); (3) The CRC did not record the information carefully; (4) The IP vendor did not provide documents as required; (5) The carrier personnel did not operate in a standardized manner; and (6) The sponsor did not provide documents as required.

### Corrective actions

The team recommends implementing both general and step-specific corrective actions to overcome the above identified failure modes (Table 2). Among them, drawing the operation flowcharts for each step, producing pocket-sized manuals, and enhancing the training of IDS pharmacy staff and investigators may be the most important general actions. For the drug dispensing phase, which was associated with the highest-risk failure modes, the corrective actions formulated by the team mainly include the following: IDS pharmacy staff should strengthen the review of whether prescriptions comply with the protocol, implement inventory reminder measures, mark in advance which trials require review of randomization forms during IP dispensing, and embed barcode technology in the drug management system.

## Discussion

An IDS pharmacy plays a pivotal role in supporting clinical trial research by ensuring the safe and efficient IP management. Appropriate risk management can significantly improve the safety of participants in clinical trials and the reliability of the trial results.^16^ This study represents the inaugural application of FMEA to an IDS pharmacy, and to the best of the authors’ knowledge, no previous research has been published on this topic. Within this study, the IP management processes within the IDS pharmacy were optimized, leading to a comprehensive analysis of all potential failure modes that could impact participant safety, the integrity of trial data, and the regulatory compliance of clinical trials. High-risk failure modes were identified, and corrective actions were proposed. This study offers a valuable reference for IP risk management in clinical trials at other sites in the future.

One of the most significant research findings was the identification that the failure modes with the highest risks are the inconsistency between the prescription information and the randomization form information during the drug dispensing process, as well as the discovery of insufficient drug inventory during the follow-up of participants. Randomized allocation is widely regarded as the optimal design for evaluating the efficacy of new therapies.^17^ The randomization form is generated by the IRT system based on specific randomization methods. Therefore, the randomization form is the most crucial basis for the drug administration process. It is a document that records the specific information of each participant assigned to the different treatment groups (such as the experimental group and the control group) and the drug allocation. Investigators usually write prescriptions for participants based on the randomization form. If there is an inconsistency between the prescription and the information on the randomization form (such as the participant number, drug number, or drug quantity), it will directly lead to incorrect medication for the participant, exerting a huge impact on the safety of the participant and the reliability of the trial results. Thus, when dispensing drugs in a randomized trial, pharmacy staff should strictly check the consistency of the drug information with that on the prescription and the randomization form, then calculate the drug dosage to ensure the accuracy of medication in the clinical trial.^4^ The insufficient inventory of drugs during participants’ follow-up is also a very serious problem, which will directly lead to the interruption of participants’ medication. The risk of insufficient inventory is higher in trials requiring manual ordering, and whether open-label and double-blind trials involve manual ordering depends on the sponsor’s inventory management settings. In addition, even if inventory management is automatically controlled by the IRT system, insufficient inventory may occur if the enrollment at the research site exceeds the expectation and the highest inventory warning level (periodic automatic replenishment level) set in the IRT system is insufficient to cope with the higher enrollment. Therefore, to ensure the medication needs of all participants during each follow-up, we recommend: For trials not using an IRT automatic control system, pharmacy staff should set reminders based on the inventory quantity in the pharmacy’s CTMS, and remind the CRA to replenish the inventory for trials with low inventory during the monthly inventory check. For trials using an IRT automatic control system, pharmacy staff should clarify the highest inventory warning level set in the IRT with the CRA at the start of the trial, and remind the CRA to modify the inventory warning level as soon as possible when the number of participants enrolled exceeds the expectation.

In this study, 60.0% (6/10) of the top 10 items with the highest RPN scores and 62.5% (5/8) of those with the highest severity score of 5 points originated from the drug dispensing process. This study shows that apart from the issues of randomization forms, prescriptions, and inventory, which have been identified as high-risk factors in the above analysis, incorrect drug dispensing by pharmacy staff is also an important aspect that deserves attention in the drug dispensing process. This is mainly related to IP packaging in clinical trials. The most commonly mentioned features are that the outer packaging is usually black and white and contains small print, along with the unique drug numbers for IPs. In addition, the outer packaging may sometimes contain information in multiple languages or, conversely, have incomplete labels, such as missing data on the drug name, drug specification, expiration date, or specific protocol information.^18^ In addition, these drugs are often sound-alike/look-alike drugs. This creates opportunities for potential errors in selecting the incorrect product during the dispensing process and may lead to the drugs intended for one participant being given to another. Some commonly used corrective actions include attaching barcodes to the outer packaging upon receipt of each shipment and using barcode scanning as a double-check safety measure when the drugs are dispensed.^1,18^ It should be noted that barcodes are affixed through manual processes by pharmacy staff, which may introduce human operation errors. Therefore, drug dispensing must be based on the verification of the information on the drug packaging. Barcode scanning should only serve as a double-check tool to reduce errors, rather than being the sole verification process for dispensing. At the same time, labels containing participant information can be attached to the outer packaging, or the participant’s random number, initials, and visit period can be written with a permanent marker to ensure medication safety.^18^ However, these corrective actions also impose a significant additional workload on pharmacy staff. To ensure the medication safety of participants, the IDS pharmacy should be adequately staffed.

In addition, 37.5% (3/8) of the failure modes with the highest severity score of 5 points in this study were related to the drug storage condition. This is mainly because the control and monitoring of the storage conditions for IPs are crucial for maintaining the integrity of the products and the safety of the participants. The pharmacy staff must ensure that the temperature storage conditions of the products can be recorded at all times while IPs are managed by the pharmacy. At the same time, it should also be confirmed that each device and temperature monitoring device has been calibrated within the calibration date range specified for the device, and that calibration documents shall be available for inspection.^1^ The recording of drug storage conditions mainly needs to focus on 2 aspects: (1) the shipment of drugs from the supplier to the IDS pharmacy and (2) the storage period in the IDS pharmacy. The high risks identified in this study regarding the storage conditions mainly come from the shipment of drugs from the supplier to the pharmacy. This is mainly due to concerns about the logistics personnel, as the shipment standards for IPs are higher than those for commercial drugs. Therefore, it is recommended that 2 pharmacy staff members be involved in the verification work during the product receiving process. In this study, the problem of the storage condition in the IDS pharmacy was not identified as a failure mode. It is speculated that the main reason may be that this site has set up short message service (SMS) and telephone alarm reminders for the cold chain monitoring system and, in addition, has narrowed the alarm temperature range compared with the required drug storage temperature (for example, the temperature requirement for drugs in the refrigerated area is 2-8 °C, and the alarm temperature range is set to 3-7 °C). In this way, when there is a significant change in the ambient temperature, pharmacy staff can take measures in advance to avoid temperature deviations.

Another point to be mentioned is that the type of IP in the prescription and its usage, dosage, or treatment duration not adhering to the trial protocol, as well as the quantity of the IP prescribed not covering the participant’s visit window period based on the usage and dosage, have also been observed as potentially highly erroneous. In clinical trials, investigators may not be familiar with the protocol-defined dose calculation or dose-rounding requirements.^19^ It has been reported that prescribing error rates in general anticancer studies were 1.1% to 5.36%.^19-23^ Nonetheless, since prescription errors in clinical trials may have a greater impact on patient safety and may determine the success or failure of the trial, a more stringent standard with an error rate approaching zero is required.^24^ Studies have shown that active interventions taken by pharmacy staff, such as discussing the prescription method with investigators before the start of the study and providing a prescription guide via email to investigators and clinical research coordinators, can improve the safety of IPs used in clinical trials and promote compliance with clinical trial protocols.^19^

FMEA has been previously proven effective by numerous studies, although the process can be complex and time-consuming. Nonetheless, FMEA is well suited for analyzing many healthcare processes. The ultimate goal of FMEA is to prevent harm from reaching a patient. Reducing the frequency of errors, making errors more obvious, and reducing the severity of the impact of an error can make systems safer.^8,14^

The notable strength of this study is the successful implementation of the FMEA model, which, through the proactive engagement of pharmacy staff, enabled the comprehensive identification and determination of all, or nearly all, potential failure modes and their underlying causes in the management of clinical trial medications within the IDS pharmacy setting. Furthermore, this study systematically identified high-risk failure modes and formulated targeted corrective actions to mitigate these risks. The proactive application of FMEA not only enhances the vigilance of pharmacy staff in their daily practice but also encourages their active involvement in minimizing medication-related discrepancies. By adopting this approach, the study aims to optimize participant safety and ensure the highest standards of research quality, ultimately striving to reduce the clinical trial error rate to as close to zero as possible.

### Limitations

The major limitation of FMEA stems from its inherent subjectivity and potential inaccuracies in the identification of failure modes and the quantification of associated risks. This is largely due to its reliance on brainstorming, where team-based estimations form the foundation for establishing evaluation criteria, as the primary research technique. However, this study addresses these limitations by not only adhering to the practical standards mandated by relevant Chinese regulations but also incorporating comprehensive references to IP management guidelines from countries such as the United States and Canada. Given that the fundamental requirements for IP management processes are largely consistent across various countries, there is potential to address the aforementioned limitations effectively. Moreover, since the primary objective of such analyses is to prioritize failures based on their criticality rather than relying solely on quantitative metrics, the RPN value itself is of secondary importance. In practice, it is essential to focus on each identified failure mode comprehensively to ensure robust risk management and mitigate potential vulnerabilities.

Furthermore, prior studies have highlighted the failure to perform a secondary FMEA following the implementation of corrective actions as an additional limitation of this type of research.^13,25^ Nevertheless, the results of this study indicate that FMEA can effectively identify potential failure modes within the IDS pharmacy management process. By employing FMEA, pharmacy staff were able to conduct an in-depth exploration of nearly all conceivable failure modes, a process that fosters enhanced critical thinking and vigilance in future routine practices.

## Conclusion

Through the proactive engagement of pharmacy staff, the FMEA methodology was successfully applied to identify and prioritize potential failure modes within the IDS pharmacy management process. This approach facilitated the formulation of targeted corrective actions, thereby safeguarding participant safety and continuously improving the quality of clinical trials as well as IP management.

## Data Availability

The original contributions presented in the study are included in the article. Further inquiries can be directed to the corresponding author.

## Additional information

The authors submitted the following information regarding their contributions to this work. Drafted the work or revised it critically for important intellectual content: B. Zhang; investigation, data curation: M. Song, Z. Wang; oversaw the entire project, and approved the final version of the manuscript: X. Pang, Y. Zhou.

## References

[1] Kay SC, Luke DG, Tamer HR. ASHP guidelines for the management of investigational drug products. Am J Health-Syst Pharm. 2018;75(8):561–573. doi:10.2146/ajhp170812 29626006

[2] Amin SR, Avila JG, Boron MJ, et al HOPA investigational drug service best practice standards. Hematology/Oncology Pharmacists Association. Accessed March 10, 2025. https://www.hoparx.org/documents/109/HOPA16_IDS_Guidelines.reviewed_2018.pdf

[3] Amin S, Polley S, DeFrates S, et al National Comprehensive Cancer Network investigational drug service consensus recommendations. Am J Health-Syst Pharm. 2022;79(6):486–491. doi:10.1093/ajhp/zxab455 34849539

[4] Siden R, Tamer HR, Skyles AJ, Weadock S, Redic K. Pharmacist-prepared dispensing guidelines for drugs used in clinical research. Am J Health-Syst Pharm. 2012;69(12):1021–1026. doi:10.2146/ajhp110082 22644977

[5] Song K, Yu M, McLuckie R, et al Development of complexity categories for an investigational drug services complexity scoring tool to assess pharmacy effort in clinical trial initiation and maintenance. Am J Health-Syst Pharm. 2023;80(21):1557–1563. doi:10.1093/ajhp/zxad138 37335865

[6] Kane MP, Fessele K, Gordilis-Perez J, et al Medication safety in cancer clinical trials: an analysis of medication error reports at a comprehensive cancer center. J Clin Oncol. 2007;25:6547.

[7] International Council for Harmonisation of Technical Requirements for Pharmaceuticals for Human Use (ICH) . ICH E6(R3) Guideline for good clinical practice. Published 2025. Accessed March 10, 2025. https://database.ich.org/sites/default/files/ICH_E6%28R3%29_Step4_FinalGuideline_2025_0106.pdf

[8] Institute for Safe Medication Practices Canada . *The Systems Approach to Quality Assurance for Pharmacy Practice: A Framework for Mitigating Risk*. Published 2012. Accessed March 10, 2025. https://abpharmacy.ca/wp-content/uploads/QAFramework_web.pdf

[9] Anjalee JAL, Rutter V, Samaranayake NR. Application of failure mode and effect analysis (FMEA) to improve medication safety: a systematic review. Postgrad Med J. 2021;97(1145):168–174. doi:10.1136/postgradmedj-2019-137484 32843483

[10] Lin S, Wang N, Ren B, et al Use of failure mode and effects analysis (FMEA) for risk analysis of drug use in patients with lung cancer. Int J Environ Res Public Health. 2022;19(23):15428. doi:10.3390/ijerph192315428 36497503 PMC9739421

[11] Jain K. Use of failure mode effect analysis (FMEA) to improve medication management process. Int J Health Care Qual Assur. 2017;30(2):175–186. doi:10.1108/IJHCQA-09-2015-0113 28256927

[12] Hertig JB, Hultgren KE, Parks S, et al Development and assessment of a medication safety measurement program in a long-term care pharmacy. Consult Pharm. 2016;31(2):112–118. doi:10.4140/TCP.n.2016.112 26842689

[13] Anjalee JAL, Rutter V, Samaranayake NR. Application of failure mode and effects analysis (FMEA) to improve medication safety in the dispensing process-a study at a teaching hospital, Sri Lanka. BMC Public Health. 2021;21(1):1430. doi:10.1186/s12889-021-11369-5 34284737 PMC8293514

[14] Stojković T, Marinković V, Jaehde U, et al Using failure mode and effects analysis to reduce patient safety risks related to the dispensing process in the community pharmacy setting. Res Social Adm Pharm. 2017;13(6):1159–1166. doi:10.1016/j.sapharm.2016.11.009 28011161

[15] American Society for Healthcare Risk Management . Strategies and tips for maximizing failure mode and effect analysis in an organization. J Healthc Risk Manag. 2002;22(3):9–12. doi:10.1002/jhrm.5600220304 17342987

[16] Cancellieri G, Provenzani A, Polidori C, et al Risk assessment of clinical trial protocols: a tool for hospital pharmacists to reduce human error in experimental drug management. Eur J Hosp Pharm. 2025;32(2):121–125. doi:10.1136/ejhpharm-2024-004154. 39009418

[17] Lim CY, In J. Randomization in clinical studies. Korean J Anesthesiol. 2019;72(3):221–232. doi:10.4097/kja.19049. Published correction appears in *Korean J Anesthesiol*. 2019;72(4):396. doi:10.4097/kja.19049.e1 30929415 PMC6547231

[18] Cruz JL, Brown JN. Safety risks with investigational drugs: pharmacy practices and perceptions in the Veterans Affairs health system. Ther Adv Drug Saf. 2015;6(3):103–109. doi:10.1177/2042098615584924 26240744 PMC4519741

[19] Moon JY, Lee Y, Han JM, et al Effects of pharmacist interventions on reducing prescribing errors of investigational drugs in oncology clinical trials. J Oncol Pharm Pract. 2020;26(1):29–35. doi:10.1177/1078155219834723 30832556

[20] Vantard N, Ranchon F, Schwiertz V, et al EPICC study: evaluation of pharmaceutical intervention in cancer care. J Clin Pharm Ther. 2015;40(2):196–203. doi:10.1111/jcpt.12242 25594148

[21] Han JM, Ah YM, Suh SY, et al Clinical and economic impact of pharmacists’ intervention in a large volume chemotherapy preparation unit. Int J Clin Pharm. 2016;38(5):1124–1132. doi:10.1007/s11096-016-0339-9 27365091

[22] Nerich V, Borg C, Villanueva C, et al Economic impact of prescribing error prevention with computerized physician order entry of injectable antineoplastic drugs. J Oncol Pharm Pract. 2013;19(1):8–17. doi:10.1177/1078155212447974 22623276

[23] Suzuki S, Chan A, Nomura H, Johnson PE, Endo K, Saito S. Chemotherapy regimen checks performed by pharmacists contribute to safe administration of chemotherapy. J Oncol Pharm Pract. 2017;23(1):18–25. doi:10.1177/1078155215614998 26561587

[24] Fell GL, O’Loughlin AA, Nandivada P, et al Methods to reduce medication errors in a clinical trial of an investigational parenteral medication. Contemp Clin Trials Commun. 2016;4:64–67. doi:10.1016/j.conctc.2016.06.005 27489888 PMC4967555

[25] Shebl NA, Franklin BD, Barber N. Failure mode and effects analysis outputs: are they valid? BMC Health Serv Res. 2012;12:150. doi:10.1186/1472-6963-12-150 22682433 PMC3405478

